# Predictive value of serum iron on heart failure in patients with acute ST‐segment elevation myocardial infarction

**DOI:** 10.1002/clc.23990

**Published:** 2023-02-13

**Authors:** Wen Chen, Guoli Lin, Caizhi Dai, Kaizu Xu

**Affiliations:** ^1^ Department of Cardiology, The Affiliated Hospital of Putian University Putian University Fujian China

**Keywords:** acute ST‐segment elevation myocardial infarction, heart failure, serum iron

## Abstract

**Background:**

In clinical practice, heart failure often occurs after acute myocardial infarction, and a new biomarker for its early prediction is urgently needed. The aim of this study was to investigate the relationship between serum iron and heart failure after acute ST‐segment elevation myocardial infarction (STEMI).

**Methods:**

A total of 41 patients with heart failure after STEMI and 31 controls were included in the study. The demographic variables and baseline clinical characteristics of both groups were analyzed.

**Results:**

There were no significant differences between patients with heart failure and controls in terms of demographic characteristics. There were significant differences in terms of serum iron, N terminal pro‐B‐type natriuretic peptide levels, left atrial diameter, and left ventricular ejection fraction. Binary logistic regression analyses demonstrated that serum iron (odds ratio [OR]: 0.804, 95% confidence interval [CI]: 0.699–0.924) and Tn‐I (OR: 1.072, 95% CI: 1.011–1.137) were independent predictors for heart failure (*p* < .05, respectively). Receiver operating characteristic analysis showed that the area under the curve for serum iron was 0.808 (95% CI: 0.707–0.908, *p* < .01). The best cutoff value of serum iron was 11.87 μmol/L (sensitivity: 87.1%; specificity: 68.3%).

**Conclusions:**

Patients with heart failure after STEMI have lower serum iron levels than patients without heart failure after STEMI. Serum iron levels are a risk factor for heart failure after STEMI.

## Graphical abstract

Different levels of serum iron in ST‐segment elevation myocardial infarction.

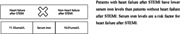

## 1 INTRODUCTION

Heart failure (HF) is a serious and terminal stage of cardiovascular disease, with high morbidity and mortality. With the aging of the population, the prevalence of HF is also increasing, and is increasingly becoming an important global public health problem.^1^ Myocardial infarction (MI) is an important risk for HF. There is a high incidence of postinfarction HF in China but insufficient information on the epidemiology. Currently, the prognosis of patients with postinfarction HF has improved, but all‐cause mortality, cardiovascular event rates, and rehospitalization rates remain high, which is related to the severity of the disease and whether there is timely diagnosis and treatment of these patients.^2^ Iron catalyzes redox reactions that maintain cell balance or drive toxic damage. It not only plays a central role in cardiac energetics, but can also cause the death of cardiac muscle cells through uncontrolled oxidative stress.^3^ One study directly analyzed myocardial tissue samples from patients with and without HF and found that myocardial iron levels were lower in patients with HF. This was associated with reduced mitochondrial enzyme activity in HF patients, suggesting that iron plays a key role in myocardial function.^4^ Patients with HF combined with iron deficiency have deteriorated muscle function due to reduced mitochondrial iron stores in myocytes, resulting in decreased cardiac function.^5^ The incidence of HF after acute MI is increasing. Clinical observation found that the occurrence of HF was more than 24 h after acute MI. Iron catalyzes redox reactions that maintain cell balance or drive toxic damage. It not only plays a central role in cardiac energetics, but can also cause the death of cardiac muscle cells through uncontrolled oxidative stress.^3^ One study directly analyzed myocardial tissue samples from patients with and without HF and found that myocardial iron levels were lower in patients with HF. This was associated with reduced mitochondrial enzyme activity in HF patients, suggesting that iron plays a key role in myocardial function.^4^ Patients with HF combined with iron deficiency have deteriorated muscle function due to reduced mitochondrial iron stores in myocytes, resulting in decreased cardiac function.^5^ It had been shown that reduced serum iron levels in patients with acute HF are associated with poor prognosis, including 12‐month mortality.^6^ Ye Gang et al. showed that A lower admission serum iron level is an independent predictor of acute HF in STEMI patients during hospitalization.^7^ The study also shown that low serum iron is an independent predictor of poor prognosis for acute decompensated HF regardless of hemoglobin or ferritin levels.^8^ Determining whether serum iron levels can predict HF after STEMI could improve early recognition and management. The incidence of HF after acute MI is increasing. Clinical observation found that the occurrence of HF was more than 24 h after acute MI. Therefore, sensitive indicators are needed to assess risk factors for HF before onset. The aim of our study was to determine the association of serum iron with HF in patients with acute ST‐segment elevation MI.

## 2 METHODS

### 2.1 Study design and populations

Patients with acute ST‐segment elevation myocardial infarction (STEMI) hospitalized in the Department of Cardiovascular Medicine from December 2021 to May 2022 were selected. STEMI patients with new onset of HF during hospitalization were the HF group. STEMI patients with no HF during hospitalization were control group. Exclusion criteria included: Pre‐existing HF; a history of previous infarction; recent acute infection or chronic inflammatory disease; malignant tumor; valvular heart disease; anemia; restrictive and hypertrophic cardiomyopathy; currently taking iron medication and Hepatic disease. The diagnosis of acute ST‐segment elevation MI was in compliance with the guidelines for the diagnosis and treatment of acute ST‐segment elevation MI published by the Chinese Medical Association in 2019 (Supporting Information: 1).^9^ The diagnosis of HF after MI mainly depended on the history, symptoms, signs and auxiliary examination, and was in line with the criteria of the 2020 Expert Consensus on the Prevention and Treatment of Heart Failure After Myocardial Infarction (Supporting Information: 2).^1^

### 2.2 Data collection

All data were obtained from the hospital's digital information system. Baseline demographic and clinical characteristics of all patients were recorded, including information on age, gender, hypertension, and diabetes. The normal range of each indicator. Serum iron (12–30 μmol/L), N terminal pro‐B‐type natriuretic peptide (NT‐pro‐BNP) levels (300–1800 Pg/mL), total cholesterol (TC) (1.6–.17 mmol/L), high‐density lipoprotein cholesterol (HDL‐C) (1.16–1.55 mmol/L), low‐density lipoprotein cholesterol (LDL‐C) (2.6–3.4 mmol/L), uric acid (155–357 μmol/L), alanine aminotransferase (ALT) (7–40 IU/L), aspartate aminotransferase (AST) (13–40 IU/L), hemoglobin (130–175 g/L), d‐dimer (0–0.5 μg/mL), c‐reactive protein (CRP) (0–10 mg/L), troponin I (Tn‐I) (0–0.03 ng/mL).

### 2.3 Clinical measurements

On admission, blood samples were placed through an anterior axillary venipuncture into an ethylenediaminetetraacetic acid‐treated test tube or a plain test tube. Complete blood counts were measured using an automated hematological analyzer (CAL8000; Mindray Corporation). The levels of NT‐pro‐BNP and Tn‐I were measured using an automated fluorescent immunoassay (Getein1600; Geteinbiotech). Blood biochemical examination and serum iron were measured using an automated biochemical analyzer (CM‐800; Geteinbiotech).

### 2.4 Echocardiography

Echocardiography was performed in all patients using a Vivid 7 echocardiography device (General Electric). All patients underwent two‐dimensional, M‐mode, and Doppler echocardiography.

### 2.5 Statistical analysis

The Kolmogorov–Smirnov test was used to demonstrate the normality of the included variables, and *p* > .05 was defined as normally distributed data. Continuous variables, expressed as mean ± standard deviation, were compared between two normally distributed continuous variables using the independent samples *t* test, while the Mann–Whitney *U* test was used to compare differences between non‐normally distributed continuous variables. Categorical variables, expressed as frequencies and percentages, were compared using *χ* ^2^ tests. Binary logistic regression analysis was used to identify potential independent associations between postinfarction HF and clinical parameters. Receiver operating characteristic (ROC) curves were analyzed to identify the best cutoff values for the prediction of postinfarction HF. *p* < .05 represents a statistically significant difference (two‐tailed test). All statistical analyses were conducted using the Statistical Package for Social Sciences software (SPSS 19.0 for Windows; IBM).

## 3 RESULTS

### 3.1 Patient characteristics

A total of 41 patients with HF (80.5% males; mean age: 64.49 ± 12.09 years) and 31 controls (70.9% males; mean age: 63.84 ± 9.83 years) were included in the study. The demographic and clinical characteristics of the patients are summarized in Table 1. There were no significant differences between patients with HF and controls in terms of demographic characteristics.

**Table 1:** Clinical and demographic properties of two groups.

Variables	Heart failure (*n* = 41)	Control (*n* = 31)	*p*
Age (years)	64.49 ± 12.09	63.84 ± 9.83	.808
BMI (kg/m^2^)	24.6 ± 3.86	24.4 ± 3.90	.542
Male gender (%)	33 (80.5%)	22 (70.9%)	.407
Hypertension (%)	26 (63.4%)	17 (54.8%)	.637
Diabetes mellitus (%)	18 (43.9%)	7 (22.5%)	.069
Culprit lesions			
LM (%)	1 (2.4%)	1 (3.2%)	.679
LAD (%)	16 (39%)	8 (25.8%)	.315
LCX (%)	16 (39%)	13 (41.9)	.813
RCA (%)	8 (19.5%)	9 (29%)	.407
Smoker (%)	20 (48.8%)	15 (48.4%)	.581
Onset‐to‐ED (min)	425.36 ± 169.79	410.96 ± 171.80	.724
D‐to‐W (min) (min)	68.51 ± 10.36	66.35 ± 9.76	.373

Abbreviations: BMI, body mass index; D‐to‐W, door‐to‐wire; LAD, left anterior descending artery; LCX, left circumflex artery; LM, left main coronary artery; Onset‐to‐ED, onset‐to‐emergency department; RCA, right coronary artery.

### 3.2 Comparison of clinical and echocardiographic parameters between the two groups

There were significant differences in serum Iron (11.35 ± 5.58 vs. 16.01 ± 4.26), NT‐pro‐BNP levels (4883.17 ± 7695.15 vs. 493.32 ± 360.39), left anterior descending artery (39.98 ± 7.24 vs. 35.97 ± 5.04), and left ventricular ejection fraction (52.03 ± 10.74 vs. 58.43 ± 7.35) between the HF and control group. There were no significant differences in terms of TC, HDL‐C, LDL‐C, uric acid, ALT, AST, hemoglobin, d‐dimer, CRP, peak Tn‐I (Table 2).

**Table 2:** Comparison of laboratory parameters and echocardiography parameters in heart failure and control groups.

Variables	Heart failure (*n* = 41)	Control (n = 31)	*p*
TC (mmol/L)	4.66 ± 1.53	4.25 ± 1.08	.184
HDL‐C (mmol/L)	1.01 ± 0.17	1.11 ± 0.31	.090
LDL‐C (mmol/L)	3.17 ± 1.35	2.81 ± 0.92	.206
Uric acid (μmol/L)	417.35 ± 134.54	438.98 ± 103.88	.406
ALT (IU/L)	37.67 ± 26.57	30.95 ± 23.51	.268
AST (IU/L)	52.63 ± 49.31	42.23 ± 35.71	.303
Serum iron (μmol/L)	11.35 ± 5.58	16.01 ± 4.26	.001
Hemoglobin (g/L)	141.97 ± 14.27	137.42 ± 15.66	.203
d‐dimer (μg/mL)	1.08 ± 1.46	0.84 ± 1.12	.476
CRP (mg/L)	14.86 ± 16.91	12.08 ± 27.38	.616
Peak NT‐pro‐BNP (pg/ml)	4883.17 ± 7695.15	493.32 ± 360.39	.002
Peak Tn‐I (ng/ml)	14.57 ± 10.98	10.31 ± 9.41	.088
LAD (mm)	39.98 ± 7.24	35.97 ± 5.04	.044
LVEF (%)	52.03 ± 10.74	58.43 ± 7.35	.007

Abbreviations: ALT, alanine aminotransferase; AST, aspartate aminotransferase; CRP, C‐reactive protein; HDL‐C, high‐density lipoprotein cholesterol; LAD, left atrial diameter; LDL‐C, low‐density lipoprotein cholesterol; LVEF, left ventricular ejection fraction; Nt‐pro‐BNP, N terminal pro B type natriuretic peptide; TC, total cholesterol; Tn‐I, troponin I.

### 3.3 Predictors of HF

Binary logistic regression analyses demonstrated that serum iron and Tn‐I were independent predictors for HF (*p* < .05, respectively) (Table 3).

**Table 3:** binary logistic regression analysis to detect the independent predictors of heart failure.

Variables	*β*	Wals	*p*	OR	95% CI
Serum iron	−.219	9.360	.002	0.804	0.699–0.924
Tn‐I	.069	5.335	.021	1.072	1.011–1.137
LAD	−.063	1.578	.209	1.065	0.965–1.176
Age	.001	0.001	.987	1.001	0.949–1.055

Abbreviations: CI, confidence interval; LAD, left atrial diameter; OR, odds ratio: Tn‐I, troponin I.

### 3.4 ROC curve

**Figure 1:**
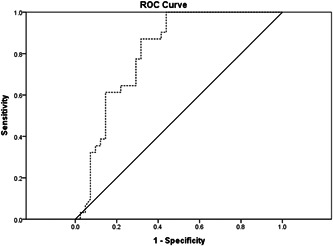
Receiver operating curve characteristics of serum iron for predicting heart failure.

For the prediction of HF, ROC analysis showed that the area under the curve for serum iron was 0.808 (95% confidence interval [CI]: 0.707–0.908, *p* < .01). The best cutoff value of serum iron was 11.87 μmol/L (sensitivity: 87.1%; specificity: 68.3%) (Figure 1).

## 4 DISCUSSION

This paper is a retrospective case–control study looking at the predictive value of serum iron levels on HF after STEMI involving 41 patients at a single medical center in China. Our study showed that serum iron levels were lower in patients with HF than in controls, but the left atrium was larger than in controls. Serum iron and Tn‐I were independently associated with HF. In addition, ROC analysis revealed that the best cutoff value of serum iron to predict HF occurrence was 11.87 μmol/L with a sensitivity of 87.1% and a specificity of 68.3%.

There was relatively little information on the epidemiology of postinfarction HF in China. The BRIGHT study found that the incidence of HF on admission in patients with acute infarction who underwent emergency PCI was 14.3%, of whom 88% were STEMI patients.^10^ Data from the China PEACE study showed that the incidence of in‐hospital early onset HF in STEMI patients was decreasing from 2001 to 2011, but was still 12.7%.^2^ Myocardial cell loss was an important cause of cardiac remodeling and the development of HF after infarction. Cardiomyocytes in the ischemic zone activated apoptotic signaling pathways in response to oxidative stress, inflammatory response, and other injury factors, which in turn mediated cardiomyocyte apoptosis.^11^ Iron was involved in the synthesis and function of a variety of enzymes and proteins involved in oxygen transport and storage, electron transfer, and oxidation–reduction.^12^ Iron deficiency leaded to a reduction in mitochondrial oxidative capacity.^13^ Decreased cardiac function may be caused by a systemic iron deficiency that compromises the cardiac energy metabolism.^14^ Studies had shown that patients with HF had reduced myocardial iron levels, leading to decreased myocardial function.^15^ Iron had also been shown to be involved in the pathogenesis of atherosclerotic coronary artery disease.^16^ Free iron promoted the oxidation of low‐density lipoprotein. Uptake of low‐density lipoprotein by low‐density lipoprotein receptors on macrophages leaded to the recruitment of foam cells, whose infiltration and necrotic core expansion were key to coronary atherosclerosis.^17^ Serum iron concentration was a measure of circulating iron bound to transferrin and serves as a proxy for iron status. Serum iron may not be as easily stored as serum ferritin, but it is a measure of iron supply to bone marrow and other tissues, and is one of the primary biochemical indicators of iron status. Moreover, serum iron had shown a strong negative correlation between iron concentrations and cardiovascular disease.^18^ Several studies had shown that serum iron levels may be a good marker for short‐term risk of cardiovascular disease in older adults.^19^, ^20^, ^21^ The PREDIMED trial shown that, among Spanish adults aged 55–80 years at high cardiovascular disease risk, low iron concentrations in serum were associated with increased short‐term risk of cardiovascular disease, and these associations were stronger in women.^22^ Although the relationship between serum iron deficiency and myocardial iron deficiency is not fully understood, systemic iron deficiency was observed in two of the last three patients with HF.^14^ Moreover, iron deficiency in MI predicts a poor prognosis.^23^ In patients with a first anterior ST‐segment elevation MI, iron deficiency was associated with larger infarcts, more extensive microvascular obstruction, and higher frequency of adverse left ventricular remodeling. The possible mechanisms were iron deficiency decreased endothelial nitric oxide synthase/soluble guanylate cyclase/protein kinase G pathway activity associated with oxidative/nitrosative stress and increased infarct size after transient coronary occlusion.^24^

There are some limitations in this article. First, This study is a retrospective study, and the population is small. Second, the interventional approach to acute MI may have an impact on the prognosis of HF. Third, whether basal serum iron levels in patients are associated with HF needs further confirmation. Finally, further large‐scale prospective studies are needed to clarify the role of serum iron in predicting HF after acute MI.

## 5 CONCLUSIONS

Patients with HF after STEMI have lower serum iron levels than patients without HF after STEMI. Serum iron levels are a risk factor for HF after STEMI. Though the study is small, it could lead to further studies on the relationship between iron levels, cardiovascular events, and decisions on management.

## Supporting information

Supporting information.

Supporting information.

## Data Availability

Data available on request to the corresponding author due to privacy/ethical restrictions.

## References

[1] Branch of Cardiovascular Physicians, Chinese Medical Doctor Association, China Cardiovascular Health Alliance, The Expert Consensus Working Group on the Prevention and Treatment of Heart Failure After Myocardial Infarction . Expert consensus on the prevention and treatment of heart failure after myocardial infarction. Chin Circ J. 2020;35(12):1166‐1189.

[2] Li J , Li X , Wang Q , et al. ST‐segment elevation myocardial infarction in China from 2001 to 2011 (the China PEACE‐Retrospective Acute Myocardial Infarction Study): a retrospective analysis of hospital data. Lancet. 2015;385(9966):441‐451. 24969506 10.1016/S0140-6736(14)60921-1 PMC4415374

[3] Dharmakumar R , Nair AR , Kumar A , Francis J . Myocardial infarction and the fine balance of iron. JACC Basic Transl Sci. 2021;6(7):581‐583. 34368506 10.1016/j.jacbts.2021.06.004 PMC8326290

[4] Melenovsky V , Petrak J , Mracek T , et al. Myocardial iron content and mitochondrial function in human heart failure: a direct tissue analysis. Eur J Heart Fail. 2017;19(4):522‐530. 27647766 10.1002/ejhf.640

[5] Mistry R , Hosoya H , Kohut A , Ford P . Iron deficiency in heart failure, an underdiagnosed and undertreated condition during hospitalization. Ann Hematol. 2019;98(10):2293‐2297. 31402406 10.1007/s00277-019-03777-w

[6] Jankowska EA , Kasztura M , Sokolski M , et al. Iron deficiency defined as depleted iron stores accompanied by unmet cellular iron requirements identifies patients at the highest risk of death after an episode of acute heart failure. Eur Heart J. 2014;35(36):2468‐2476. 24927731 10.1093/eurheartj/ehu235

[7] Ye G , Liu L , Yu J , Gan F , Wei HC . [Predictive value of serum iron level for in‐hospital acute heart failure after acute ST‐elevated myocardial infarction]. Nan Fang Yi Ke Da Xue Xue Bao. 2015;35(4):610‐614. 25907956

[8] Ueda T , Kawakami R , Nogi K , et al. Serum iron: a new predictor of adverse outcomes independently from serum hemoglobin levels in patients with acute decompensated heart failure. Sci Rep. 2021;11(1):2395. 33504934 10.1038/s41598-021-82063-0 PMC7840917

[9] Cardiovascular Branch of Chinese Medical Association, Editorial Board of Chinese Journal of Cardiovascular Disease . Guidelines for the diagnosis and treatment of acute ST‐segment elevation myocardial infarction. Chin J Cardiovasc Dis. 2019;41(10):766‐783.

[10] Han Y , Guo J , Zheng Y , et al. Bivalirudin vs heparin with or without tirofiban during primary percutaneous coronary intervention in acute myocardial infarction: the BRIGHT randomized clinical trial. JAMA. 2015;313(13):1336‐1346. 25775052 10.1001/jama.2015.2323

[11] Teringova E , Tousek P . Apoptosis in ischemic heart disease. J Transl Med. 2017;15(1):87. 28460644 10.1186/s12967-017-1191-y PMC5412049

[12] Beard JL . Iron biology in immune function, muscle metabolism and neuronal functioning. J Nutr. 2001;131(2S‐2):568S‐580S. 11160590 10.1093/jn/131.2.568S

[13] Pozzo J , Fournier P , Delmas C , et al. Absolute iron deficiency without anaemia in patients with chronic systolic heart failure is associated with poorer functional capacity. Arch Cardiovasc Dis. 2017;110(2):99‐105. 28189387 10.1016/j.acvd.2016.06.003

[14] Ponikowski P , Kirwan BA , Anker SD , et al. Ferric carboxymaltose for iron deficiency at discharge after acute heart failure: a multicentre, double‐blind, randomised, controlled trial [published correction appears in Lancet. 2021 Nov 27;398(10315):1964]. Lancet. 2020;396(10266):1895‐1904. 33197395 10.1016/S0140-6736(20)32339-4

[15] Miñana G , Cardells I , Palau P , et al. Changes in myocardial iron content following administration of intravenous iron (Myocardial‐IRON): study design. Clin Cardiol. 2018;41(6):729‐735. 29607528 10.1002/clc.22956 PMC6489786

[16] Cornelissen A , Guo L , Sakamoto A , Virmani R , Finn AV . New insights into the role of iron in inflammation and atherosclerosis. EBioMedicine. 2019;47:598‐606. 31416722 10.1016/j.ebiom.2019.08.014 PMC6796517

[17] Meng H , Wang Y , Ruan J , et al. Decreased iron ion concentrations in the peripheral blood correlate with coronary atherosclerosis. Nutrients. 2022;14(2):319. 35057500 10.3390/nu14020319 PMC8781549

[18] Das De S , Krishna S , Jethwa A . Iron status and its association with coronary heart disease: systematic review and meta‐analysis of prospective studies. Atherosclerosis. 2015;238(2):296‐303. 25544180 10.1016/j.atherosclerosis.2014.12.018

[19] Ekblom K , Marklund SL , Jansson JH , Hallmans G , Weinehall L , Hultdin J . Iron stores and HFE genotypes are not related to increased risk of first‐time myocardial infarction: a prospective nested case‐referent study. Int J Cardiol. 2011;150(2):169‐172. 20447705 10.1016/j.ijcard.2010.04.001

[20] Hsu HS , Li CI , Liu CS , et al. Iron deficiency is associated with increased risk for cardiovascular disease and all‐cause mortality in the elderly living in long‐term care facilities. Nutrition. 2013;29(5):737‐743. 23352175 10.1016/j.nut.2012.10.015

[21] Mørkedal B , Laugsand LE , Romundstad PR , Vatten LJ . Mortality from ischaemic heart disease: sex‐specific effects of transferrin saturation, serum iron, and total iron binding capacity. The HUNT study. Eur J Cardiovasc Prev Rehabil. 2011;18(5):687‐694. 21450624 10.1177/1741826710390134

[22] Gutierrez‐Bedmar M , Olmedo P , Gil F , et al. Low serum iron levels and risk of cardiovascular disease in high risk elderly population: nested case‐control study in the PREvención con DIeta MEDiterránea (PREDIMED) trial. Clin Nutr. 2021;40(2):496‐504. 32591250 10.1016/j.clnu.2020.05.044

[23] Dharmakumar R , Nair AR , Kumar A , Francis J . Myocardial infarction and the fine balance of iron. JACC Basic Transl Sci. 2021;6(7):581‐583. 34368506 10.1016/j.jacbts.2021.06.004 PMC8326290

[24] Inserte J , Barrabés JA , Aluja D , et al. Implications of iron deficiency in STEMI patients and in a murine model of myocardial infarction. JACC Basic Transl Sci. 2021;6(7):567‐580. 34368505 10.1016/j.jacbts.2021.05.004 PMC8326269

