# Supplementary material for: Predictive value of serum iron on heart failure in patients with acute ST‐segment elevation myocardial infarction
Source: Clin Cardiol. 2023 Feb 13;46(4):449–53. doi: 10.1002/clc.23990 (PMC10106665; doi:10.1002/clc.23990)
Supplement: Supplementary file 1 — Supporting information. [file CLC-46-449-s002.docx]

The criteria for diagnosis of acute STEMI

1.symptoms: severe crushing pain (usually over 10-20 min) in the retrosternal or precordial region, radiating to the left upper arm, jaw, neck, back or shoulder; often accompanied by nausea, vomiting, profuse sweating and dyspnea.

2.Physical examination: The patient's vital signs should be closely monitored. Observe the general state of the patient, whether there is wet and cold skin, pale face, irritability, jugular vein anger, etc. auscultate for pulmonary rales, arrhythmias, heart murmurs and gallop rhythm.

3.Electrocardiogram: the characteristic electrocardiogram of STEMI is ST-segment elevation (monophasic curve) with or without pathological Q-wave and R-wave depression (ST-segment changes can be insignificant in positive posterior wall myocardial infarction), often with mirror image ST-segment depression in the corresponding leads.

4.Serological and imaging tests: serum cardiac troponin (cTn) is elevated and/or decreases and is at least 1 time above the upper limit of normal (99th percentile of the upper reference value); coronary angiography or intracavitary imaging or autopsy confirms coronary thrombosis.

**References**

Cardiovascular Branch of Chinese Medical Association, Editorial Board of Chinese Journal of Cardiovascular Disease. Guidelines for the diagnosis and treatment of acute ST-segment elevation myocardial infarction.*Chinese Journal of Cardiovascular Disease.*2019;41(10):766-783.
