# Supplementary material for: Predictive value of serum iron on heart failure in patients with acute ST‐segment elevation myocardial infarction
Source: Clin Cardiol. 2023 Feb 13;46(4):449–53. doi: 10.1002/clc.23990 (PMC10106665; doi:10.1002/clc.23990)
Supplement: Supplementary file 2 — Supporting information. [file CLC-46-449-s001.docx]

The criteria for diagnosis of heart failure

1.Symptoms: orthopnea or Paroxysmal nocturnal dyspnea;

2.Signs: pulmonary rales or jugular venous filling or bilateral lower limb oedema or lateral shift or diffusion of the apical beats;

3.Natriuretic peptide test: different NT-proBNP diagnostic cut-off values are stratified according to age and renal function. NT-proBNP >450 pg/ml (<50 years), 900 pg/ml (50-75 years), 1800 pg/ml (>75 years); renal insufficiency (glomerular filtration rate <60 ml/min) >1200pg/ml.This diagnostic criteria has been added to the supplemental material.

**References**

Branch of Cardiovascular Physicians, Chinese Medical Doctor Association, China Cardiovascular Health Alliance, The Expert Consensus Working Group on the Prevention and Treatment of Heart Failure After Myocardial Infarction. 2020 Expert Consensus on the Prevention and Treatment of Heart Failure After Myocardial Infarction.*Chinese Circulation Journal.* 35(12):1166-1189.
